# Changes in the Provision of Institutionalized Mental Health Care in Post-Communist Countries

**DOI:** 10.1371/journal.pone.0038490

**Published:** 2012-06-08

**Authors:** Adrian P. Mundt, Tanja Frančišković, Isaac Gurovich, Andreas Heinz, Yuriy Ignatyev, Fouad Ismayilov, Miklós Péter Kalapos, Valery Krasnov, Adriana Mihai, Jan Mir, Dzianis Padruchny, Matej Potočan, Jiří Raboch, Māris Taube, Marta Welbel, Stefan Priebe

**Affiliations:** 1 Department of Psychiatry and Psychotherapy, Charité Campus Mitte, Universitätsmedizin Berlin, Berlin, Germany; 2 Department of Psychiatry and Psychological Medicine, School of Medicine, University of Rijeka, Rijeka, Croatia; 3 Moscow Research Institute of Psychiatry, Moscow, Russia; 4 Department of Communication Skills, Medical Psychology and Psychotherapy, Kazakh National Medical University, Almaty, Kazakhstan; 5 Department of Psychiatry, Azerbaijan Medical University, Baku, Azerbaijan; 6 Theoretical Biology Research Group, Budapest, Hungary; 7 Department of Psychiatry, University of Medicine and Pharmacy, Tg Mures, Romania; 8 Republican Scientific and Practical Centre for Mental Health, Minsk, Belarus; 9 Department of Psychiatry, Charles University, Prague, Czech Republic; 10 Department of Psychiatry, Riga Stradin’s University, Riga, Latvia; 11 Institute of Psychiatry and Neurology, Warsaw, Poland; 12 Unit for Social and Community Psychiatry, Barts and The London School of Medicine and Dentistry, Queen Mary University of London, London, United Kingdom; University of Adelaide, Australia

## Abstract

**Background:**

General psychiatric and forensic psychiatric beds, supported housing and the prison population have been suggested as indicators of institutionalized mental health care. According to the Penrose hypothesis, decreasing psychiatric bed numbers may lead to increasing prison populations. The study aimed to assess indicators of institutionalized mental health care in post-communist countries during the two decades following the political change, and to explore whether the data are consistent with the Penrose hypothesis in that historical context.

**Methodology/Principal Findings:**

General psychiatric and forensic psychiatric bed numbers, supported housing capacities and the prison population rates were collected in Azerbaijan, Belarus, Croatia, Czech Republic, East Germany, Hungary, Kazakhstan, Latvia, Poland, Romania, Russia and Slovenia. Percentage change of indicators over the decades 1989–1999, 1999–2009 and the whole period of 1989–2009 and correlations between changes of different indicators were calculated. Between 1989 and 2009, the number of general psychiatric beds was reduced in all countries. The decrease ranged from −11% in Croatia to −51% in East Germany. In 2009, the bed numbers per 100,000 population ranged from 44.7 in Azerbaijan to 134.4 in Latvia. Forensic psychiatric bed numbers and supported housing capacities increased in most countries. From 1989–2009, trends in the prison population ranged from a decrease of −58% in East Germany to an increase of 43% in Belarus and Poland. Trends in different indicators of institutionalised care did not show statistically significant associations.

**Conclusions/Significance:**

After the political changes in 1989, post-communist countries experienced a substantial reduction in general psychiatric hospital beds, which in some countries may have partly been compensated by an increase in supported housing capacities and more forensic psychiatric beds. Changes in the prison population are inconsistent. The findings do not support the Penrose hypothesis in that historical context as a general rule for most of the countries.

## Introduction

In 1939, Lionel Sharples Penrose (1898–1972) described for the first time inverse relationships between psychiatric bed numbers and the size of the prison population analysing data from 18 European countries [1]. The so called Penrose hypothesis or Penrose law suggests that decreasing psychiatric bed numbers are related to an increase in the prison population. Even though this hypothesis may be a simplification, the issue continues to be of importance [2], [3] and has been replicated and discussed in different settings [4]–[6]. One study questions this relationship for high-income countries and postulates a direct, not inverse, relationship of psychiatric bed numbers and prison populations for low- and middle-income countries (LMICs) [7]. The direct relationship was explained by a lack of all resources for either kind of institution in LMICs and a positive association of the indicators with economic measures and development. Nevertheless, it has been suggested that severely mentally ill and other people who fall below a certain level of adaptation to societal norms have a risk for voluntary or involuntary institutionalisation.

In 2002, it was estimated for the United States that the number of people with mental disorders in prisons was more than two and a half times the number of people with mental disorders in all psychiatric hospitals of the country combined [8]. The rate of the severely mentally ill in prisons was estimated to be 15% of the prison population [9]. People with mental disorders in prisons are considered such a substantial problem that a number of diversion programs have been initiated [10]–[12]. The police, other enforcing agencies and the legal justice systems often fail to identify people with mental disorders [13]. Psychiatric and forensic psychiatric bed numbers, the prison population and places in supported housing services have been suggested as indicators of institutionalized mental health care of people with mental disorders [14].

From the 1950s to the 1970s, most Western countries underwent psychiatric reforms, establishing community based mental health services and supported residential housing opportunities for people with persistent mental disorders [15]. A driving force of the reforms was a growing belief that long-term hospitalization in psychiatry would be incompatible with human rights and societal values stipulating the inclusion of all individuals into community life [16]. Other factors included the notion that asylums provided a non-therapeutic environment leading to inactivity and withdrawal of patients, and that community based services might provide more effective treatments [17]. The intention to reduce costs by replacing expensive hospital based care through cheaper services in the community may also have played a role. There has been a debate as to what degree community based services can compensate for reducing psychiatric hospital services [18].

In various countries, intensified outpatient treatment, acute home treatment and assertive outreach have been established to reduce the need for inpatient treatment [19]. In the literature, the term “revolving door patient” was coined for people with chronic mental disorders requiring repeated inpatient treatment in spite of the availability of intensified community based services [20]. The use of illicit drugs or so called “antisocial behaviour” can cause the exclusion of persons with chronic mental disorders from community based programmes [21]. A “revolving door phenomenon” has been described not only for patients in psychiatric hospitals, but also for people with mental disorders who are repeatedly imprisoned for minor criminal charges [22]. Patients with first-admission psychosis had a 9% risk for one or multiple incarcerations in a prospective follow-up study in the United States [23]. In most Western European countries, the prison population and forensic bed numbers have been increasing whereas general psychiatric bed numbers have continuously decreased [24]. Those findings have caused a debate whether in Western Europe there is a trend towards trans- or re-institutionalization of people with mental disorders [14].

Before the political changes of 1989, in the Soviet Union and in Romania, psychiatric services had been used for the treatment of dissidents [25]. In the Soviet Union, there had been an increase of the bed numbers for that reason [26]. Post-Soviet psychiatry is facing the challenge to re-define the professional identity and to build a service system that meets the need of rapid social, economic and political changes [27]. The Penrose-Law may be limited at times of massive political and societal change, given the fact that it merely takes into account quantitative measures of capacities, not the composition of the population within a given institution (e. g. mentally ill, criminal or healthy dissident). The composition of a population within a given institution may shift following significant political/societal change. Nonetheless, in this historical context, it is important to form a research collaboration that evaluates current trends of institutionalization to have a starting point for future discussions on possible trans- or re-institutionalization of mentally ill.

Mental health care in Central and Eastern European countries (CEEC) had been influenced by Soviet psychiatry, albeit to a varying degree. For example in Poland, mental health service provision developed more similarly to worldwide trends: between 1970 and 1990, before the political change, psychiatric bed numbers were reduced by about 20% [28]. After the political change of 1989, all CEEC underwent important reforms of mental health care provision including the reduction of psychiatric hospital beds, reforms of mental health legislation and transformation of the reimbursement systems [29].

Decisions to increase or reduce psychiatric bed numbers may largely be politically motivated in most countries. Research evidence on actual trends and influencing factors, however, might inform political decisions. The present study aimed to provide evidence on trends of institutionalised mental health care in post-communist countries. More specifically, we investigated to what degree indicators of institutionalized mental health care such as general psychiatric bed numbers, forensic bed numbers, prison population numbers and capacities in supported housing services for mentally ill were altered after the political change in 1989, and tested whether the findings were consistent with the Penrose hypothesis.

The findings of this study may have implications for health and social policy, particularly on mental health service development, and for mental health service research. An international and interdisciplinary discussion should follow on how societies can best balance the inclusion of all individuals with mental disorders and deal with behaviour that may require institutionalization.

## Methods

### Procedures

The research was conducted by a network of researchers that was established for this study. Experts in psychiatry and public mental health were contacted in post-communist CEEC and former Soviet countries. Selection procedure: First, experts with personal contacts to either the Charité Universitätsmedizin Berlin or the Queen Mary University of London through former research collaborations were invited to participate for their countries [30], [31]. New members of the network were recruited by reference of other members (snowballing). For all remaining countries in the region, we invited experts through emails in the English language based on their international publication record, if there were publicly available publications in the English language and email addresses. All collaborators in the study were medical doctors with a background in psychiatry or clinical psychologists. They were asked to provide data on indicators of institutionalized mental health care for their entire country. No funding could be offered to the international collaborators for the data collection. If one expert declared that most of the requested data (more than half of the data points) were not available or two experts did not respond to the request, the country was excluded from the study. Experts from twelve countries in the region responded to the request and contributed data sets to the research network from the following countries: Azerbajan, Belarus, Croatia, Czech Republic, East Germany, Hungary, Kazakhstan, Latvia, Poland, Romania, Russia and Slovenia. Experts from two countries responded to the request willing to contribute, but could not come up with the required data. Experts from three countries declared that they were not able to retrieve the required information. The contacted experts from the remaining countries did not respond to the request.

### Definition of the Indicators

General psychiatric bed numbers include all inpatient services in general adult psychiatry and child- and adolescent psychiatry, but not day hospital services and psychosomatic or psychotherapeutic rehabilitation wards. Forensic beds were preferred over the number of forensic treatment cases. In some countries, however, forensic cases are treated in general psychiatric hospitals on an individual basis and no fixed number of beds is assigned to forensic services. The number of forensic treatment cases was taken as indicator only if the number of beds was not available. Prison population rates include pretrial detainees and convicted offenders. The supported housing services include services for chronically mentally ill, for mentally disabled (if separate from physically disabled), for persons with chronic substance use, homes and communities for mentally ill and various forms of protected accommodation schemes [32]. Homes for old people and dementia facilities were not taken into account. Definitions of each indicator were refined during the process of data collection in order to acquire comparable data between the countries.

### Data Sources

Primary national data sources were preferred over internationally available data lists. The data sources include National Institutes of Mental Health, Ministries of Health or Social Welfare and National Prison Administrations. For more detailed information on the data sources in each country, please, refer to a supplementary document (Annex S1). International data sources as the International Centre for Prison Studies were used if no national data sources on prison population rates were available [33]. If there was no national or international data source available, we tried to obtain data for a specified region of the country (e.g. a metropolitan region or a federal state or a county).

### Analyses

Changes between 1989 and 1999, between 1999 and 2009, and between 1989 and 2009 were calculated in percentages. We analysed the trends for uniformity between the countries and for trends towards harmonization of the range of values between the countries. Most analyses were descriptive and exploratory. To determine whether changes from 1989–2009 in general psychiatric bed numbers correlated with changes in any of the other indicators of institutionalization, we used Spearman’s rank correlations. For the correlation with supported housing services we only took changes from 1999–2009 into account to have a sufficient number of observations. P-values of <.05 were considered statistically significant; n.s.  =  not significant.

## Results

### General Psychiatric Bed Numbers

In the first decade after the political change from 1989–1999, psychiatric bed numbers decreased in all 12 participating countries. In the later decade from 1999–2009, in the Eastern part of Germany, Kazakhstan and Poland, we found an increase in psychiatric bed numbers. In all other countries, the capacities were further reduced. Looking back over the past two decades from 1989–2009, a decrease in psychiatric bed numbers occurred in all countries ranging from 11% in Croatia to 51% in the Eastern part of Germany. In the year 2009, the psychiatric bed numbers per 100,000 population ranged from 44.7 in Azerbaijan to 134.3 in Latvia (Table 1).

**Table 1:** Indicators of institutionalized mental health care in twelve Central and Eastern European countries.

		Azerbai-jan	Belarus	Croatia	Czech Republic	Germany (East)	Hungary	Kazakh-stan	Latvia	Poland	Romania	Russia	Slovenia
Psychiatric bedsper 100.000	1989	73.4	116.6	106.7 (1995)	140.0 (1990)	156.3	123.0	126	203.6 (1991)	96.0	95.4 (1990)	133.7	80.9
	1999	50.3	102.0	95.6 (2001)	112.9	64.7	100.3	90	184.4	74.4	88.3 (1998)	119.8	77.3
Change in %	1989–1999	−32	−13	−10	−19	−59	−19	−29	−9	−23	−7	−10	−4
	2009	44.7	70.0	95.5 (2008)	103.3	77.2	75.5	92	134.3	79.3	74.8	108.0	65.6
Change in %	1999–2009	−11	−31	0	−9	19	−25	2	−27	7	−15	−10	−15
Total change in %	1989–2009	−39	−40	−11	−26	−51	−39	−27	−34	−17	−22	−19	−19
Forensic treatments andspecialised bedsper 100.000	1989	1.7	n. a.	n. a.	15.3 (1990)	2.7 (1993)	1.9	n. a.	n. a.	1.5	7.2	5.0	n. a.
	1999	4.0	5.5	n. a.	7.2	6.7	1.8	n. a.	1.7	0.8	7.2	10.3	n. a.
Change in %	1989–1999	135			−54	148	−5			−47	0	106	
	2009	3.6	6.4	7.8 (2008)	3.5	13.2	1.9	n. a.	0.7	3.8	7.2	12.1	1.2
Change in %	1999–2009	−10	16		−51	97	6		−59	375	0	18	
Total change in %	1989–2009	112			−77	389	0			153	0	142	
Prison population rates per 100.000	1989	n. a.	327.0 (1992)	n. a.	216	189.5	151.9	358 (1992)	386.8 (1996)	153.6	171 (1992)		55.8
	1999	291	620.0 (1998)	50 (2000)	224	81.6	150.4	548 (1998)	368.8	146.9	200 (1998)	676.9 (2001)	49.3
Change in %	1989–1999		90		4	−57	−1	53	−5	−4	17		−12
	2009	240	468.0 (2006)	93 (2008)	207	80.4	153.3	382	312.9	219.6	133	585.9	65.5
Change in %	1999–2009	−18	−25	86	−8	−2	2	−30	−15	50	−34	−13	33
Total change in %	1989–2009		43		−4	−58	1	7	−19	43	−22		17
Supported housing services per 100.000	1989	n. a.	108.6	n. a.	n. a.	n. a.	75.1 (1992)	n. a.	n. a.	80.7	n. a.	85.4 (1990)	n. a.
	1999	n. a.	114.5	205.5 (2003)	61.9 (2007)	50.2 (2000)	78.0	n. a.	n. a.	95.5	n. a.	91.1	39.8
Change in %	1989–1999		5				4			18		7	
	2009	18.2	120.4	218.1	108.8	62.4	86.4	n. a.	218.9	109.5	n. a.	85.6	48.9
Change in %	1999–2009		5	6	76	24	11			15		−6	23
Total change in %	1989–2009		11				15			36		0	

Indicators of institutionalized mental health care are reported for the years 1989, 1999 and 2009 if not indicated otherwise. Missing data points are indicated as n. a.  =  not available. Percentage changes are shown for the decades 1989–1999 and 1999–2009 as well as for the changes over the twenty-year period 1989–2009 following the political change in 1989.

### Forensic Psychiatric Bed Numbers

Forensic psychiatric bed numbers, or treatment cases, increased over both decades in East Germany, Russia and over the last decade in Belarus and Poland, as well as in the decade following the political change in Azerbaijan. They decreased over both decades in the Czech Republic, over the last decade in Azerbaijan and Latvia. They remained unchanged in Romania and Hungary. The numbers range from 0.7 per 100,000 population in Latvia to 13.2 in the Eastern part of Germany. Over the past two decades from 1989–2009, five countries had an increase of forensic psychiatric bed numbers, most pronounced in East Germany by 389%. Only in the Czech Republic there was a continuous decrease, in total by −77% (Table 1).

### Prison Population

Over the two decades following the political change, there was an increase in the prison population in Belarus, Hungary, Kazakhstan, Poland, and Slovenia. A decrease in prison population rates over the two decades is shown for the Czech Republic, East Germany, Latvia and Romania. For Azerbaijan, Croatia and Russia, we could only obtain data for the second decade after the political change. They show a decrease of the prison population in Azerbaijan by −18% and in Russia by −13% from high numbers in 1999 and an increase in Croatia by 86% from low numbers.

There was no uniformity regarding changes of the prison population neither for trends nor for absolute numbers. Rates of the prison population per general population differ considerably between lowest rates in the former Yugoslavian countries and East Germany to higher rates in other Eastern European countries to highest rates in former Soviet countries. In the past decade there was a trend towards harmonization between the countries with a decrease in countries with high rates and an increase in countries with low rates (Table 1).

### Supported Housing Capacities

Data on the capacities for supported housing ranged from 18.4 per 100,000 population in Azerbaijan to 218.1 in Croatia. In countries with available data for both decades, Belarus, Hungary, Poland showed an increase in supported housing capacities. In Russia, they remained unchanged. In a majority of countries, namely Belarus, Croatia, Czech Republic, Germany, Hungary, Poland and Slovenia, an increase of supported housing capacities was found over the past decade. The most pronounced increase of 76% in recent years occurred in the Czech Republic. Only Russia showed a decrease over the past decade. No data on supported housing services were available from Romania and Kazakhstan (Table 1).

### Associations of Changes between Different Indicators

We did not find any significant correlations between the indicators of institutionalized care over time. The changes in general psychiatric bed numbers did neither correlate significantly with changes in prison population rates (r = .28; p = .46; n.s.; 1989–2009) nor with changes in forensic treatment places (r = −.04; p = .93; n.s.; 1989–2009) nor with changes in supported housing capacities (r = .45; 0 = .26; n.s.; 1999–2009).

## Discussion

The strength of the paper is the establishment of a research collaboration for evaluating possible indicators of institutionalized mental health care covering a range of Eastern European and post-Soviet countries. The study reveals trends of institutional capacities and service provision in the face of a unique historic situation requiring fundamental changes in social policy.

### Main Findings

Uniform trends over the past two decades were seen for a decrease of general psychiatric hospital bed numbers in all countries and for an increase of supported housing services in most countries. Trends in both directions were observed for the prison population and forensic treatment capacities. De-hospitalization from general psychiatric hospitals occurred in all countries in the post-communist era, most pronounced in the decade directly after the political change in 1989–1999. In most of the countries, de-hospitalization continued in the past decade. It has come to a halt in three out of 12 countries in the past decade. A possible trend for harmonization may have started for prison population rates in the past decade with those countries having very high rates showing a decrease. There was no general support for the Penrose hypothesis as far as it is applicable for this historical context.

### Interpretation and Comparison Against the Literature

The decrease of general psychiatric beds may not have caused an increase of the prison population, as a general statistical phenomenon for most countries undergoing post-communist changes. However, the data neither rule out the Penrose hypothesis for single countries nor exclude a possible trans-institutionalization of people with mental disorders from psychiatric hospitals to prisons. Before the political change, prison capacities were high and in some countries used for political dissenters [26]. Due to the release of political dissenters and due to permissive politics including amnesties in the post-revolutionary years, some countries experienced a steep drop in the prison population during the years after the political change (e.g. East Germany and Czech Republic). This does not necessarily show in the ten-year intervals that we chose for the presentation of the data (e.g. Czech Republic): after the steep drop in the post-revolutionary years, the prison population had gradually increased to the level of before 1989 again, raising the question as to whether people with mental health problems, especially with drug addiction [34], may have replaced dissidents in prisons.

The reduction of bed capacities in psychiatric institutions may have been a uniform political paradigm for the region, whereas policies regarding the development of forensic capacities range from massive expansion in East Germany [35] and Russia to massive reductions in the Czech Republic. Those trends may reflect opposite political assumptions about the attribution of psychopathology to criminal behaviour. Decreasing psychiatric hospital bed numbers were shown as a worldwide phenomenon in the recently published Mental Health Atlas, WHO 2011 [36]. The strongest decrease was observed for the American and European regions. The authors of the World Mental Health Atlas do not provide an interpretation as to whether this is a success reflecting an underlying shift towards effective community mental health care or a way of reducing funding in mental health care or both. In several CEEC, there has been a trend towards decentralisation and privatisation of service delivery [37]. It has been described for Russia, and it may hold true for several other post-Soviet countries [38], [39], that psychiatric services are still largely hospital based [40]. Outpatient treatment is traditionally centralized in large “dispensaries” [41]. If there has been any reduction in bed capacities, it might have been less motivated by initiatives to establish decentralized community based care but rather by a lack of funding [42]. The need for social and occupational rehabilitation after the political change still receives little consideration [43].

In a majority of CEEC, forensic psychiatric capacities have been rising. However, the capacities do not necessarily relate to the quality of treatment. In many places forensic psychiatry is still struggling to develop a professional identity, with changing mental health acts and few professional teachers and trainers. In some countries, forensic psychiatry is mostly concerned with assessment rather than treatment, and the treatment is delegated to prison health services or mental hospitals [44]. Substance use and other mental disorders are a major public mental health concern in penitentiary systems worldwide [45], [46]. Even after reforms with the intention to reduce prison population rates in Russia, the country continues with one of the highest rates in the world [47]. It was suggested that the prevalence of substance use disorders in post-Soviet countries has been increasing in both the general population and prison populations [48].

### Implications

The first implication of this study is the need for better national data collections on important indicators of institutionalization. There is a need to harmonize the definition of specific indicators to render data internationally comparable. Psychiatric bed reduction should be linked to establishing community based services. The quality of supported services needs evaluation so that patients are not subjected to similar conditions as in the old psychiatric hospitals, which have been closed down or reduced in size. Further research has to address whether the high prison population rates in former Soviet and Eastern European countries reflect high rates of mentally ill people in the penitentiary system and whether inefficient social policy or psycho-social community care may be linked to those continuously high prison population rates.

One may conclude that more accurate data are required for a reliable analysis of trends of care provision over time. Similar criticism of the limited availability and reliability of data on mental health care provision has been raised for Western Europe [24]. Thus, the limitations of the data are not unique to the Eastern Europe, whilst some of the dramatic changes shown in this study may be understood only against the background of an unusual historical period of dramatic political and societal change. Wider research considering political factors, health policies and economic data [49] are required to understand the drivers behind the different trends in institutionalized mental health care and possibly anticipate future changes.

### Limitations

Limitations of the paper may arise from the incomplete data and the difficulty to assess the quality of all data that were available. Data were gathered nationally within each country from primary sources if available and checked against public secondary data collections for plausibility if possible. We tried and agreed on uniform definitions for each indicator as far as possible. For example, the definition, of what a psychiatric bed in Germany is, posed unexpected difficulties: we tried to separate “purely psychosomatic”, “psychotherapeutic” wards and psychiatric or psychosomatic “rehabilitation” wards, because this kind of explicitly non-psychiatric yet psychotherapeutic inpatient treatment is established on a large scale only in Germany. A transformation of general psychiatric beds in psychotherapeutic or psychosomatic rehabilitation wards may obscure true trends of de-hospitalization. In a decreasing number of settings, hospitals run joint wards for neurology and psychiatry.

Missing data points for forensic beds and supported housing services were common for many countries, whilst data were more complete for psychiatric hospital beds. Also, the formal description of a service may say little about the precise nature and quality of the service. For East Germany, cases of changing the label of a hospital ward for chronic patients into a residential facility by just changing the sign on the door and cutting down on the staff have been reported. Similar instances may have occurred in other countries.

## Supporting Information

Annex S1 Detailed information on data sources for each country. f institutionalized mental health care in twelve Central and Eastern European countries.
(DOCX)
