# Supplementary material for: Changes in the Provision of Institutionalized Mental Health Care in Post-Communist Countries
Source: PLoS One. 2012 Jun 8;7(6):e38490. doi: 10.1371/journal.pone.0038490 (PMC3371010; doi:10.1371/journal.pone.0038490)
Supplement: Annex S1 — Detailed information on data sources for each country. f institutionalized mental health care in twelve Central and Eastern European countries. (DOCX) [file pone.0038490.s001.docx]

## Annex

Azerbaijan: The residential facilities include 1012 places for chronically disabled adults and 605 for mentally disabled children (<http://www.azstat.org/statinfo/healthcare/en/index.shtml>). Homes for chronically mentally ill were closed after the collapse of the Soviet Union due to inappropriate conditions. Community residential facilities for mentally ill are not available in the country. Psychiatric hospitals serve persons with acute and chronic disorders (<http://www.who.int/mental_health/evidence/azerbaijan_who_aims_report_english.pdf>).

Belarus: Data for general psychiatric bed numbers and supported housing capacities were provided by the Organizational and Methodological Department of the Republican Scientific and Practical Center for Mental Health in Minsk, Belarus. Prison population rates were retrieved from the International Center for Prison Studies ([www.prisonstudies.org](http://www.prisonstudies.org)).

Croatia: Availability of historic data on psychiatric beds starts with the last year of the Balkan war (1991-1995) in 1995. The number of psychiatric beds was retrieved from the Hrvatski zavod za javno zdravstvo (Croatian Agency for Public Health). Data for supported housing were retrieved from the annual report of the Ministry of Health and Social Welfare.

Czech Republic: Psychiatric bed numbers were retrieved from the Institute of Health Information and Statistics of the Czech Republic (Bed care: Health statistic. Vol. 1990-2009; ISSN 1211-0515). Prison population rates were retrieved from the Statistical Yearbooks of the Prison Service of the Czech Republic [online] available from [http://vscr.cz/generalni-reditelstvi-19/statistiky-a-udaje-103/statisticke-rocenky-1218/](http://www.vscr.cz/generalni-reditelstvi-19/statistiky-a-udaje-103/statisticke-rocenky-1218/) [cited 2011-02-03]. Supported housing capacities are published in the Statistical Yearbooks [online]. Prague: Ministry of Labour and Social Affairs, available from <http://www.mpsv.cz/cs/3869> [cited 2011-02-03].

Germany: After 1989, East Germany refers to East German States (Länder) without East-Berlin that is inextricably unified with West-Berlin and persons using services across the former limits. General psychiatric bed numbers were retrieved from the National Bureau of Statistics (Statistisches Bundesamt: Grunddaten der Krankenhäuser, Fachserie 12, Reihe 6.1.) and for Eastern Germany in 1989 from the report of the Federal Ministry of Health (Bundesministerium für Gesundheit (Hrsg.): Bericht zur Lage der Psychiatrie in der ehemaligen DDR, 1991). Forensic psychiatric bed numbers for Eastern Germany were obtained from the Ministries of Social affairs and Health of the Federal States (personal communication with ministries of all eastern Federal states (Länder)). Prison population rates were retrieved from the National Bureau of Statistics (Statistisches Bundesamt: Rechtspflege; Statistisches Bundesamt: Rechtspflege, Fachserie 10, Reihe 4.2). For the German Democratic Republic in the year 1989 the data were retrieved from the National Archives (Statistische Übersichten über den Gefangenenbestand, BArch, DO 1/3709) [[1](#_ENREF_1)]. Supported housing capacities were reported by the “Bundesarbeitsgemeinschaft der überörtlichen Sozialhilfeträger” BAGüS and Con_sens, Hamburg (BAGüS/Con_sens: Kennzahlenvergleich der überörtlichen Träger der Sozialhilfe, 2010, and personal communication of Hans-Peter Schütz-Sehring from Con_sens, consultant and specialist for people with disabilities). For the supported housing, only those Federal States were taken into account, that delivered consistent data for all years: Sachsen and Sachsen-Anhalt.

Hungary: Data for general psychiatric bed numbers and prison population rates were retrieved from the National Bureau of Statistics. There is only one Forensic Mental Institute serving the entire country.

Kazakhstan: The psychiatric bed numbers were retrieved from the Statistics collection of the Department of Psychiatry of the Kazakh National Medical University [[2](#_ENREF_2)]. Prison population rates were retrieved from the International Center for Prison Studies ([www.prisonstudies.org](http://www.prisonstudies.org)).

Latvia: General psychiatric bed numbers were retrieved from Statistical Yearbooks (Psihiskās veselības aprūpe 1991-2000; Riga, 2001; The Centre of Psychiatry; ISBN 9984-9532-0-3; <http://www.gvva.gov.lv/lv_publik/2000.pdf>) and Thematic report (Mental Health Care in Latvia 2009, Riga, 2010, The Centre of Health Economics; ISBN 977-9984-837-25-3; <http://vec.gov.lv/uploads/files/4d79e927e71cf.pdf>). Forensic bed numbers were retrieved from Yearly Health statistic reports 1999 and 2008. The prison population rate for the years 1996, 1999 and 2009 was taken from The Central Statistical Bureau of Latvia ([www.csb.gov.lv](http://www.csb.gov.lv)). Supported housing includes persons living in special social care homes for mentally ill. The rate was retrieved from official information of the Ministry of Welfare ([www.lm.gov.lv](http://www.lm.gov.lv)).

Poland: General psychiatric and forensic psychiatric bed numbers were retrieved from the Statistical Yearbooks: Mental Health Care and Neurological Care Facilities, Institute of Psychiatry and Neurology (IPiN), Department of Health Services Organisation (ZOOZ), Warszawa from the years 1990, 2000, 2010. The data for the prison population rates and for the supported housing was retrieved from the Statistical Yearbooks of the Republic of Poland, Central Institute of Statistics (Główny Urząd Statystyczny, GUS), Warszawa for the years 1990, 2000, 2009. Data for the prison population rate include pretrial detainees and convicts. The supported housing for persons with mental disorders includes capacities for chronically mentally ill and mentally disabled (including community self-help homes).

Romania: Psychiatric bed numbers were retrieved from the National Institute for Statistics. Forensic bed numbers were calculated from the websites of the four forensic psychiatric hospitals (www.spitaljebel.ro/; http://www.padurenimhhosp.ro/legaturi.html; http://www.hsapoca.ro/; <http://www.hpsihiatriestei.ro/>). The prison population rate for the years 1989 and 1999 was taken from the AxA Consulting Romania E-Step 2008, Current Education and Training Provision in Romanian Prisons, February 2008, p.9. The prison population rate for the year 2009 was retrieved from the National Institute of Statistics.

Russia: Data for the prison population was retrieved from the Russian Federal Service for the Execution of Sentences. The agency provides annual data of the prison population including pretrial detainees starting with the year 2001. We present data for the years 2001, 2006 and 2009. All the other data was retrieved from the Ministry for Public Health and Social Development.

Slovenia: Psychiatric bed numbers were retrieved from the National Institute of Public Health. Prison population rates were retrieved from the Ministry of Justice – Service for the Execution of Sentences. National statistics for forensic treatment rates do not exist. The data were retrieved from 5 out of all 6 psychiatric hospitals in the Republic of Slovenia with a catchment area of 1,292,335 persons. Seven non-governmental organizations and 6 public social welfare institutions provide supported housing. Data were retrieved from each of these 13 institutions.

1. Dölling B (2009) Strafvollzug zwischen Wende und Wiedervereinigung. Berlin: Christoph Links Verlag.

2. Altynbekov SA, Musafarov RH, Abdramankyzy A, Aimacova EM (2010) Psychiatricheskaya pomoch naseleniu respubliki Kazakhstan sa 2008-2009 gg: Statistics collection.
